# Is It Feasible to Predict Cardiovascular Risk among Healthy Vegans, Lacto-/Ovo-Vegetarians, Pescatarians, and Omnivores under Forty?

**DOI:** 10.3390/ijerph20032237

**Published:** 2023-01-27

**Authors:** Izabela Kwiatkowska, Jakub Olszak, Alicja Brożek, Anna Blacha, Marcin Nowicki, Kalina Maćkowiak, Piotr Formanowicz, Dorota Formanowicz

**Affiliations:** 1 Department of Medical Chemistry and Laboratory Medicine, Poznan University of Medical Sciences, 61-701 Poznan, Poland; 2 Institute of Computing Science, Poznan University of Technology, 60-965 Poznan, Poland

**Keywords:** cardiovascular risk, cardiovascular risk factors, biomarkers, prediction, screening, vegans, vegetarians, pescatarians, omnivores, COVID-19, pandemic

## Abstract

Guidelines for cardiovascular (CV) risk assessment among young adults are uncertain. Researchers are still looking for new tools for earlier diagnosis of cardiovascular diseases (CVD), the leading cause of mortality in the modern world. This study aimed to assess whether CV risk estimation is possible in groups of healthy individuals under the age of 40 on different dietary patterns (vegans—VEGAN (*n* = 48), lacto-/ovo-vegetarians—VEGE (*n* = 49), pescatarians—PESCA (*n* = 23), and omnivores—OMN (*n* = 35)) during the pandemic period. Four metrics containing selected risk classifiers were created, and participants were assessed using them. Groups including meat consumption showed increased CV risk predictions in the metrics assessment. The next analyzes showed statistically significant relationships between the results from the created metrics and selected non-basic biomarkers for ApoA1 (OMN group, *p* = 0.028), IL-6 (PESCA group, *p* = 0.048), HCY (VEGAN group, *p* = 0.05), and hsCRP (OMN + PESCA groups, *p* = 0.025). We found that predicting CV risk among healthy people under 40 adhering to different dietary patterns, taking into account basic and non-basic laboratory assessments and created metrics, is challenging but feasible. Furthermore, the OMN group appeared to be at the highest risk of increased CV risk in the future, while risk tended to be the lowest in the VEGAN group.

## 1. Introduction

Cardiovascular diseases (CVD) are one of the leading groups of diseases on a global scale [1,2]. About 10–20% of cardiovascular (CV) events are underlined by genetics, and 70% are due to risk factors and the consequences of an unhealthy lifestyle: hypertension, hypercholesterolemia, diabetes, and obesity, as well as environmental factors. A family history of CVD is an essential factor; however, in most cases, the family burden is a duplication of the parents’ unhealthy lifestyle, i.e., inappropriate diet and low physical activity [2,3,4,5].

Through the CV risk factors, we signify the likelihood of developing or dying from CVD within a certain period. Total CV risk is estimated based on all factors found in a person. This concept is theoretical because, in practice, it is impossible to evaluate all factors. Several hundred risk factors are known, many of which are complex, expensive, and time-consuming to measure, and probably not all factors affecting prognosis are yet known. Hence, in practice, determining the intensity of the necessary intervention (e.g., due to the patient’s lifestyle); the decision to start pharmacotherapy (e.g., in a patient with dyslipidemia or hypertension); setting treatment goals (e.g., in a patient with dyslipidemia); and patient education are based on global CV risk, which means that risk is estimated based on selected factors [2,3,4,5,6].

Numerous scales have been developed to assist in CV risk stratification that are modified depending on the population being assessed. Mainly, they include well-known (classical) risk factors, usually expressed on a 10-year scale, and are used to estimate the risk of a first fatal CV event occurring in apparently healthy individuals. Among examples, there are the Framingham CVD risk score [7], SCORE-2 (Systematic Coronary Risk Evaluation) [1,8], and PROCAM (Prospective Cardiovascular Münster Study for fatal and non-fatal myocardial infarction) [9,10]. However, these systems may underestimate risk in many cases. Recent evidence has shown that adding another factor, such as a new biomarker, can improve CV risk assessment. Therefore, some new CV risk assessment systems (Multi-Ethnic Study of Atherosclerosis—MESA or Astronaut Cardiovascular Health and Risk Modification—Astro-CHARM) have added various parameters, including, for example, high sensitivity C-reactive protein (hsCRP) serum concentration and/or coronary artery calcification [11,12]

CV risk factors can be divided into either modifiable or non-modifiable in everyday practice. There are far fewer non-modifiable factors than those we can deal with; they include male sex, age >55 years old in men and >65 years of age in women, early menopause, and family history of heart and vascular diseases. Among the modifiable factors, i.e., those that we can avoid, are (1) smoking—although awareness of the smoking impact is growing, it is still one of the leading causes of not only cardiovascular diseases but also respiratory diseases and cancers; (2) sedentary lifestyle; (3) poor diet (above all, abnormal lipid parameters (total cholesterol (TC), low-density lipoprotein cholesterol (LDL-C), high-density lipoprotein cholesterol (HDL-C), triglycerides (TG), triglyceride-rich lipoproteins as apolipoprotein B), and glucose levels being one of its consequences); (4) being overweight and obesity; (5) long-term stress can increase the risk of cardiovascular disease up to four times; (6) alcohol abuse; (7) poorly controlled hypertension; (8) poorly controlled diabetes; and others [1,13,14].

The influence of behavioral factors on health has been proven; the latest ESC guidelines from 2021 [1] put lifestyle changes as one of the main goals of managing CV risk. According to the Global Burden of Disease Study (GBD) [15], it has been estimated using the Global Health Data Exchange (GHDx) [16] that behavioral factors account for a significant proportion of mortality from CV causes (49.35%), chronic respiratory diseases (45.29%), and cancer (36.7%), with an additional estimated impact of 38.24% on general causes worldwide. Global recommendations issued by, e.g., the WHO [17,18,19], the Global Panel on Agriculture and Food Systems for Nutrition [20], the EAT-Lancet Commission Summary Report [21], or the Lancet Countdown report [22] emphasize the importance of diet and the need for changes to increase the consumption of plant-based products and reduce consumption of sources of saturated and trans fatty acids. A low-quality diet, including a high supply of products rich in saturated fatty acids (SFA) and low in polyunsaturated fatty acids (PUFA), is described as one of the most important causes of CVD [19,23,24,25,26,27].

A particularly positive effect is attributed to vegan diets, which, in terms of nutrition, are characterized not only by lower consumption of saturated fats and the lack of animal cholesterol, which is a stable of meat foods, but also the lack of high-fat dairy products and eggs. The consumption of vegetables and fruits, wholegrain products, soya products, and nuts is clearly increased, so the diet is characterized by a higher content of fiber, phytochemicals, and vitamins C and E, folic acid, and magnesium, which have beneficial health effects. The positive aspects of this diet also include a higher level of nutritional density, an increased proportion of unsaturated fatty acids, and a lower level of energy density [28].

Hence, continuous work on using new biomarkers results in new ideas, among which there are new parameters, such as lipoproteins, homocysteine, a soluble urokinase plasminogen activator receptor, inflammatory biomarkers, oxidative biomarkers, or endothelial dysfunction biomarkers [29,30,31,32,33,34,35,36,37].

This study aims to assess whether it is possible to predict CV risk among healthy people under 40, adhering to different dietary patterns using selected biochemical parameters (named basic (well-known and assessed commonly) and non-basic (assessed not routinely)). Moreover, metrics were developed based on the assessed basic laboratory parameters and data on smoking, family history of CVD, and disorders related to lipid or carbohydrate metabolism to check whether they can help assess CV risk.

An additional aim was to investigate whether selected non-basic parameters (recognized as a possible new indicator of CV risk) could be considered prognostic and helpful in the prognosis of CVD among healthy subjects under 40 years of age.

It is unique research because it tries to fill the gap in estimating CV risk among healthy people under 40 when people’s lifestyle begins to become established. The study takes into account the different dietary pattern groups. In general, research among people on vegetarian diets, including vegan ones, is a new field of science that is only just being explored. It should be emphasized that the study group is challenging because, due to its relatively young age [1] and health, it cannot be assessed using classical methods, such as Systemic Coronary Risk Estimation 2 (SCORE2), Framingham, or PROCAM.

## 2. Materials and Methods

### 2.1. Study Design

The study was conducted at the Poznan University of Medical Sciences (Department of Medical Chemistry and Laboratory Medicine) at the turn from June to July (24, 26, 29 June and 13 July 2021). The research was conducted following the recommended restrictions regarding the COVID-19 pandemic. Participants were recruited online, using social media or e-mail invitations. To recruit people on vegetarian diets, including vegan diets, advertisements were added in closed groups for people following such a diet in Poland (“Vegetarians and Vegans” with more than 24,000 members and “Vegans Poland” with 59,000 members). Participants were registered for a selected day and time out of four possible dates. The message to participants confirming participation contained the existing contraindications and instructions on preparing for the study. The first stage of the study [38] included an analysis of body composition (using the InBody analyzer (Seoul, Korea) by using the bioelectric impedance (BIA) method) and the assessment of eating habits and behavioral factors using the Food Frequency Questionnaire (FFQ) among the study groups. This form allowed for identifying nutritional patterns and finding a relationship with health outcomes [39,40,41,42,43]. The study results were thoroughly described and recently published [38]. After the analysis, the volunteers could also participate in the second part of the study involving blood tests.

Participants in the study, which included the diagnostic part, were additionally asked to complete a short questionnaire—the CV risk burden questionnaire, see Supplementary Material (Questionnaire S1), in which they were asked about the possible burden of CV risk. The questions included information on smoking and a family history of CVD and disorders related to lipid or carbohydrate metabolism.

**Figure 1:**
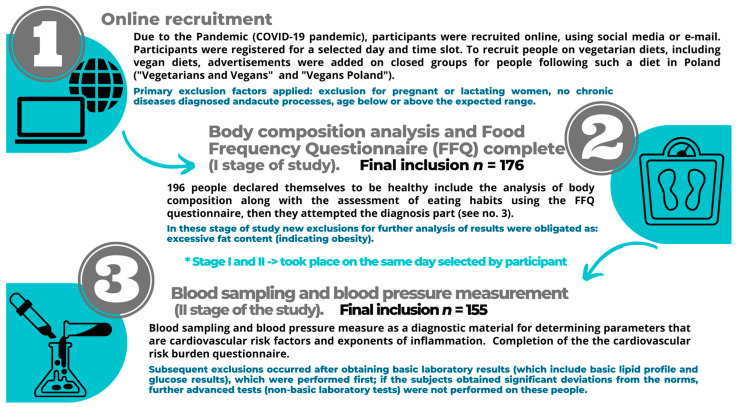
Scheme of the research process.

The research process diagram was prepared; for detailed information, see Figure 1.

### 2.2. Ethics

The study was carried out following the Declaration of Helsinki of the World Medical Association and approved by the Bioethics Committee at Poznan University of Medical Sciences (No. 237/20/2020). Each participant gave informed written consent to participate in the study.

### 2.3. Subjects

The population of this study consisted of healthy people aged 18–39, adhering to one of the following diets: (1) mixed diet/omnivores [OMN] (traditional, including meat consumption), (2) pescatarian diet [PESCA] (including the consumption of fish, eggs, and dairy products among animal products), (3) lacto-/ovo-vegetarian diet [VEGE] (eggs and dairy products including from animal products, this group includes: lacto-vegetarian, ovo-vegetarian, or lacto-ovo-vegetarian), and (4) participants on a vegan diet [vegan/-s, VEGAN] (not including meat products or products of animal origin). Using a proprietary form, the participants determined the adhering type of diet through a personal declaration. Then compliance with the FFQ questionnaire was checked (during the I stage of this study [38]). The declared dietary pattern (other than the traditional diet) should be followed for at least a year (which was an inclusion criterion). Persons entering the study were informed about the required participation in the fasting state and the factors excluding them from participation. Primary exclusion factors applied: pregnant or lactating women, chronic diseases diagnosed and acute processes, age below or above the expected range. One hundred ninety-six people declaring themselves to be healthy were recruited. They joined the study, which included the analysis of body composition along with the assessment of eating habits using the FFQ questionnaire (as the first stage of the study) and blood sampling as a diagnostic material for determining parameters that are CV factors and exponents of inflammation (as the second stage of the research). The results obtained in the first stage of the study, due to irregularities in the composition analysis (indicating obesity), showed the need to exclude 20 people (9 vegans, 7 lacto-/ovo-vegetarians, 4 pescatarians) [38]. Following exclusions occurred after obtaining primary laboratory results (which include basic lipid profile and glucose results), which were performed first; if the subjects received significant deviations from the norms or the age was above 40 (because single cases showed an age above), further analyses were not performed on these people; hence, 21 participants were excluded (5 vegans, 3 lacto-/ovo-vegetarians, 5 pescatarians, and 8 omnivores). Ultimately, 155 people participated in the study’s second stage, including non-basic determinations (a total of 41 people were excluded, and the upper age limit, due to the age criterion, was lower than in the I stage of the study; for comparison, see [38]).

The final studies based on assessing basic and non-basic parameters included 48 VEGAN, 49 VEGE, 23 PESCA, and 35 OMN.

**Figure 2:**
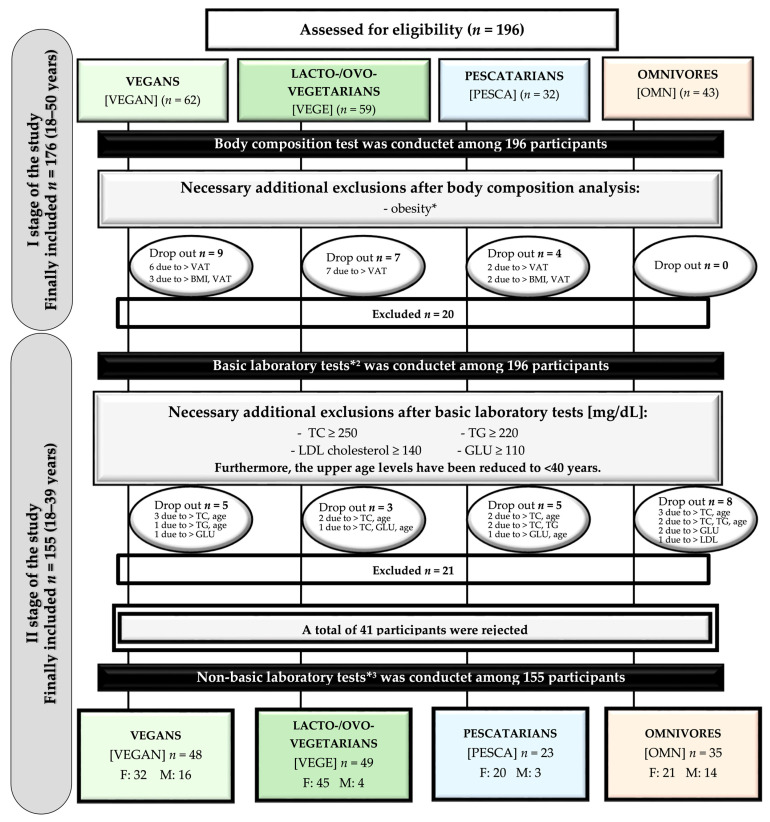
The scheme of recruiting participants. Abbreviations: F — Female, M — Male. * not every participant knew that obesity is a diseases (criteria of exclusion: Body Mass Index (BMI) ≥ 30 kg/m^2^ and/or visceral adipose tissue content (VAT) > 10 cm^2^. *^2^ basic laboratory tests included: Total cholesterol (TC) [mg/dL], Triacylglycerols (TG) [mg/dL], High-density lipoprotein cholesterol (HDL-C) [mg/dL], Low-density lipoprotein cholesterol (LDL-C) [mg/dL], Glucose (GLU) [mg/dL]. *^3^ non-basic laboratory tests included: Apolipoprotein A1 (ApoA1) [mg/dL], Apolipoprotein B (ApoB) [mg/dL], Tumor necrosis factor alpha (TNF-alpha) [ng/L], Homocysteine (HCY) [nmol/mL], Lipoprotein(a) (Lp(a)) [mg/dL], High sensitivity C-reactive protein (hsCRP) [mg/L], Interleukin 6 (IL-6) [pg/mL].

The recruitment scheme of the respondents is presented in Figure 2.

### 2.4. Biochemical Blood Tests

Biochemical analyzes were determined from venous blood after 14–16 h of fasting and collected once.

First, the basic analyses of the lipid profile (TC, TG, HDL-C, LDL-C) were determined using the Cobas b 101 (Roche brand). In contrast, glucose (GLU) levels were measured using Roche diagnostic tests and COBAS INTEGRA 400 plus. The obtained results showed the need to exclude participants whose results significantly deviated from the normal values (established exclusion criteria: TC ≥ 250 [mg/dL], TG [mg/dL] ≥ 220, LDL-C ≥ 140 [mg/dL], GLU ≥ 110 [mg/dL]), and, therefore, 41 people were eliminated (20 people due to irregularities in the first stage of the study (body composition analysis) and 21 people due to incorrect results of essential determinations).Uric acid [UA] was measured using the COBAS INTEGRA 400 plus analyzer. These biochemical parameters were assigned as basic.

Selected non-basic laboratory tests were then performed, including apolipoprotein A1 (ApoA1), apolipoprotein B (ApoB), lipoprotein(a) (Lp(a)), Tumor necrosis factor alpha (TNF-alpha), homocysteine (HCY), Interleukin 6 (IL-6) using the SunRed Enzyme-Linked Immuno-sorbent Assay (ELISA) method with the TECAN-SUNRISE reader and the Magellan software. High-sensitivity C-reactive protein (hsCRP) was determined using the DRG Diagnostics ELISA method with the TECAN-SUNRISE reader. These biochemical parameters were assigned as non-basic risk factors/biomarkers non-basic.

In addition, the following indices were calculated from the values obtained: ApoB to ApoA1 ratio (ApoB/ApoA1), TC to HDL-C ratio (TC/HDL-C), non-high-density lipoprotein (Non-HDL-C).

For the parameters ApoA, ApoB, TNF-alpha, IL-6, and hsCRP, confidence intervals [CI] were statistically calculated for each study population; see Supplementary Materials (Table S1).

It should be pointed out that studies using the ELISA method were used to compare the obtained values between groups for research purposes, so they may differ from the results obtained in commercial laboratories.

The evaluation methods of the analyzed parameters are described in the table; see Supplementary Materials (Table S1).

### 2.5. Blood Pressure Measurement

Blood pressure was measured on the subjects’ left arms, after a 15-min rest, in a sitting position, using an OMRON standardized blood pressure monitor.

### 2.6. Statistical Analysis

Statistical analysis was performed using the Jamovi statistical software [44]. Non-parametric methods were used in all analyses (the Shapiro–Wilk test confirmed the violation of normal distribution, and Levene’s homogeneity test confirmed the violation of the assumption of equal variances). To compare the results between all four groups, the Kruskal–Wallis One-Way ANOVA test was performed. Additionally, the Mann–Whitney U test was used to compare pairs of study groups. All correlations in the article were calculated using the Spearman method. The statistically significant results were set at *p* < 0.05.

## 3. Results

The study was conducted in Poland during the COVID-19 pandemic period at the Poznan University of Medical Science (Department of Medical Chemistry and Laboratory Medicine) among healthy people adhering to one diet: vegan [VEGAN], vegetarian (lacto-/ovo-vegetarian) [VEGE], pescatarian [PESCA] and traditional/mix diet—omnivores [OMN]. The average duration of adherence to the current diet among the study groups (OMN participants were omitted) was 4.0 years for VEGAN, 7.9 years for VEGE, and 4.4 years for PESCA. In addition, many VEGANs reported earlier adherence to a lacto-/ovo-vegetarian diet; therefore, the duration of meat withdrawal in this group could be longer).

### 3.1. Baseline Characteristics and the Biochemical Blood Tests

This study consisted of two stages; the first stage evaluated the subjects’ body composition analysis and an assessment of eating habits, which has been described, see [38].

The study’s second (current) stage included 155 participants (see, Figure 2 in Section 2). They were under the age of 40; in OMN, PESCA, VEGE, and VEGAN, the age was 30.0 ± 3.85; 29.3 ± 6.46; 28.2 ± 5.95; 29.6 ± 6.35 [mean ± SD years], respectively. There were no statistically significant differences in the respondents’ ages, although the similar age of participants was not controlled during the recruitment process. The recruitment to this study was mainly carried out through social media, where young adults and adults are the leading users, which could have influenced the mean age of the respondents.

Table 1 includes the participants’ lipids, lipoproteins, and other biochemical determinants and blood pressure values following four diets. Results with the gender division applied are shown for determinations where the norm is dependent on such division.

**Figure 3:**
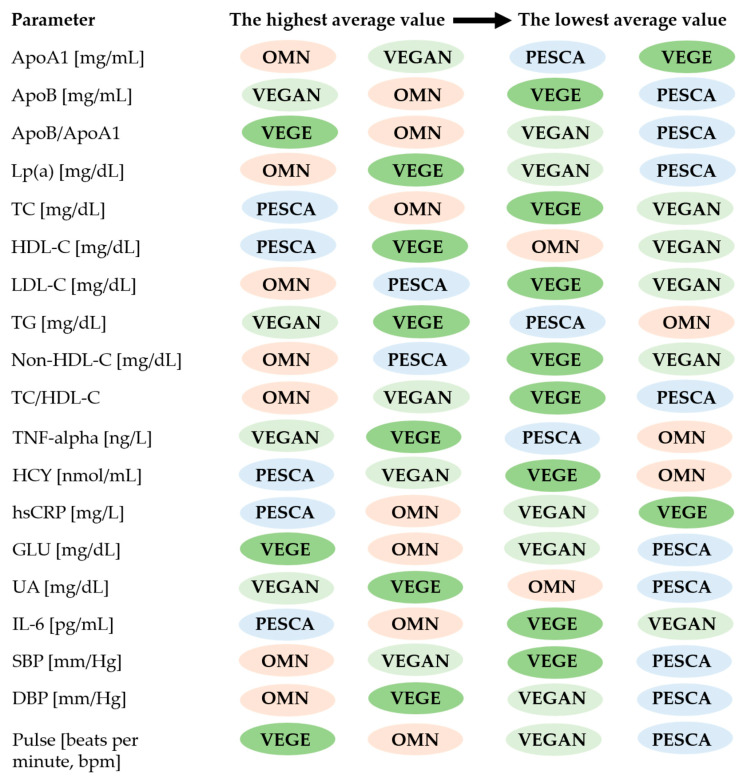
The highest and the lowest average values of biochemical parameters, pulse and blood pressure measurements in all groups marked with separate colors. Abbreviations: ApoA1—Apolipoprotein A1, ApoB—Apolipoprotein B, ApoB/ApoA1—Apolipoprotein B/apolipoprotein A1 ratio, Lp(a)—Lipoprotein(a), TC—Total cholesterol, HDL-C—High-density lipoprotein cholesterol, LDL-C—Low-density lipoprotein cholesterol, TG—Triglycerides, Non-HDL-C—Non-high-density lipoprotein cholesterol, TC/HDL-C—Total cholesterol to high-density lipoprotein cholesterol ratio, HCY—Homocysteine, TNF-alpha—Tumor necrosis factor alpha, hsCRP—High sensitivity C-reactive protein, GLU—Glucose, UA—Uric acid, IL-6—Interleukin 6, SBP—Systolic blood pressure, DBP—Diastolic blood pressure.

The summary of the results of the biochemical tests and pulse and blood pressure measurements (medium values) shown in descending order are summarized in Figure 3.

The concentrations of TC, HDL-C, LDL-C, TG, Non-HDL-C, TC/HDL-C, GLU, UA, and Lp(a) were within the recommended values; see for the recommendations [1,45,46,47,48,49,50].

Significant differences between the studied groups were observed for the following values: TC, HDL-C, and TG. The remaining parameters were checked in the population based on statistical calculations—95% confidence interval (CI), (see Supplementary Material Table S1).

### 3.2. Results of Blood Pressure Measurement

Systolic blood pressure (SBP), diastolic blood pressure (DBP), and pulse assessment results in all groups were classified as within the normal limits. The OMN group showed the highest average score for SBP and DBP among the other groups. The VEGE group showed, on average, the highest pulse values compared to the other groups; the differences in this parameter were statistically significant. The PESCA group showed, on average, the lowest SBP, DBP, and pulse values. The results are shown in Table 1.

### 3.3. Relationships between the Biochemical Analyses, Behavioral Factors, and Body Composition Parameters

Correlations analyses (see Supplementary Materials Tables S2–S5) showed statistically significant relationships between selected studied CV risk factors and inflammatory markers among one another and also concerning the body composition parameters and behavioral factors (sleep duration, physical activity) assessed in the first stage of the study [38]; however, the results were considered for only 155 participants.

As the summary of correlations measured, Table 2 shows the strongest correlations between the parameters in the study group.

The PESCA group shows, similar to the OMN group, the same strongest correlations. Furthermore, the VEGAN group’s correlation results are identical to those in the VEGE group.

### 3.4. Prediction of CV Risk

As the study participants were, on average, <40 (mean age usually 30), this age precluded the use of CV risk’s well-known schemes of estimation in all subjects, where the relevant age is at least 40 (the widely used SCORE-2); an attempt was made to assess CV risk using a different method. For this purpose, four metrics were created, taking into account the parameters of lipid and carbohydrate metabolism and the assessment of family burden.

The results of the characteristics of the metrics considered are presented in Table 3.

Each parameter included in a given metric was assigned a point scale; when the obtained value followed the metric criterion, one point was added; then the medium outcome of each group was shown. The higher the score received, the greater the estimated CV risk.

There were no statistical differences between the studied groups. Based on the obtained average values measured in each group separately, in three out of four metrics, the OMN group shows the highest estimated prognosed CV risk, while the VEGAN group in the same number (three out of four metrics) showed the lowest risk. When combining the VEGE + VEGA as meat-free groups and the OMN + PESCA as groups including meat consumption, the results showed higher prognosed CV risk using all the metrics in the OMN + PESCA.

In the next step, the results from the metrics were correlated with selected non-basic biochemical determinants (including the laboratory tests), and no statistically significant results were obtained. Then a division in the study groups was created to check possible differences between the groups taking into account the following assumptions: if none or a minority of the factors was present, a “lower value” group [L] was created (values from 0 to 1 obtained in metrics for all metrics except “Metric 2”, where a value of 0 was considered), but if the factors were present, a “higher value” group [H] was created (value > 1 obtained in metrics for all metrics except “Metric 2”, where a value of > 0 was considered). Such a scale was applied to each of the study groups separately and compared with the non-basic biochemical determinants. The results of significant differences are presented in Table 4.

In the OMN group, a lower concentration of ApoA1 (group H) seemed to increase the prognosed CV risk (measured in metric 1). Similar observations in other groups regarding the possible effect of raising the risk concern the highest concentration (in group H) of IL-6 in the PESCA group and HCY in the VEGAN group.

Groups including meat consumption (OMN + PESCA) showed significant differences concerning hsCRP in the case of its increased concentration (group H); an increased risk of CV risk could be suspected (measured in metric 2).

The concentrations of ApoA1, IL-6, HCY, and hsCRP seem to be useful new biomarkers for estimating CV risk.

**Figure 4:**
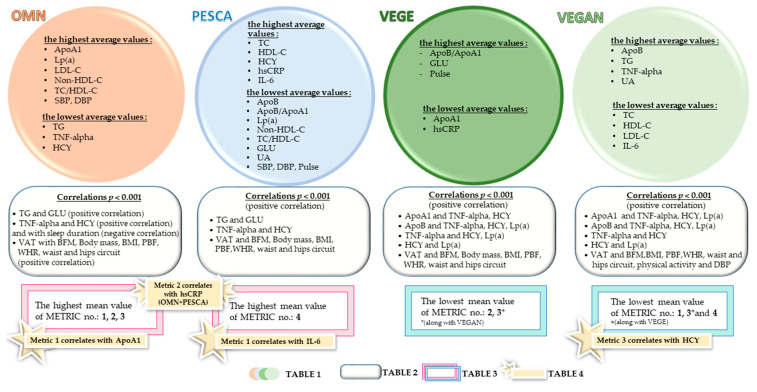
The summary of the study results. Abbreviations: Body Fat Mass (BFM) [kg], Body Mass Index (BMI) [kg/m^2^], Percentage Body Fat (PBF) [%], Skeletal Muscle Mass (SMM) [kg], Waist-Hip Ratio (WHR), Visceral Adipose Tissue(VAT) [cm^2^], as parameters from I stage of study [38]; parameters from current study (II stage): Total cholesterol (TC) [mg/dL], Triacylglycerols (TG) [mg/dL], High-density lipoprotein cholesterol (HDL-C) [mg/dL], Low-density lipoprotein cholesterol (LDL-C) [mg/dL], Non-high-density lipoprotein cholesterol (Non-HDL-C) [mg/dL], Total cholesterol to high-density lipoprotein cholesterol ratio (TC/HDL-C), Glucose (GLU) [mg/dL], Apolipoprotein A1 (ApoA1) [mg/dL], Apolipoprotein B [ApoB, mg/dL], Tumor necrosis factor alpha (TNF-alpha) [ng/L], Homocysteine (HCY) [nmol/mL], Lipoprotein(a) (Lp(a)) [mg/dL], High sensitivity C-reactive protein (hsCRP) [mg/L], Uric acid (UA) [mg/dL], Interleukin 6 (IL-6) [pg/mL], Systolic blood pressure (SBP) [mmHg], Diastolic blood pressure (DBP) [mmHG], beats per minute (bpm).

The study’s most essential results are shown schematically in Figure 4. They have been divided into four studied groups, depending on the declared diet, and the significant results have been selected and presented here.

## 4. Discussion

### 4.1. Cardiovascular Risk Factors

Comparing the obtained results of lipids and lipoproteins in the studied groups, it should be emphasized that all the results were in line with the reference values. Therefore, the comparison of differences between the obtained mean values was limited to determining possible trends.

The lipid profile assessment showed a tendency for TC and HDL-C concentrations to be significantly higher in the PESCA group when compared to other groups. On the other hand, the lowest TC and HDL-C concentrations were observed in the VEGAN group. It should be noted that HDL-C concentrations above 90 mg/dL were observed in all of the studied groups except the VEGAN group. According to the latest guidelines [9], HDL-C values above 90 mg/dL may increase the risk of atherosclerotic CVD (ASCVD).

Moreover, TG values in the VEGAN group were the highest (still within the reference range), which seems surprising, considering that people following vegan diets should reveal lower TG than the other groups. Mainly, it is due to the positive effect of products with a low supply of saturated fatty acids. On the other hand, the lowest physical activity in this group compared to the other groups (the results of the first stage of the research where behavioral factors were checked) [38] can probably explain that phenomenon. An increased habitual physical activity, a reduced total amount of carbohydrates in the diet, as well as a reduced consumption of mono- and disaccharides or the use of n-3 polyunsaturated fatty acid supplements (as recommended by the European Society of Cardiology and the European Society of Atherosclerosis (ESC/EAS)) are the factors affecting the reduction of TG concentration [45]. The results of other studies concerning TG and vegan diets are inconclusive. In Kuchta et al. [51], vegans did not show significantly lower TGs than omnivores. Similar results were obtained in a population of vegan premenopausal women compared to omnivores [52] and in a cross-sectional study by Saintila [53], where non-vegetarians had lower serum TG concentrations than vegetarians. In the Adventist Health Study-2 [54], TGs were highest in the non-vegetarian group; however, they showed lower values in the pesco-vegetarian group if compared to the vegan group, but this was not statistically significant. Other studies [55,56] have shown lower TG values among vegans compared to non-vegans and a considerable reduction in TG after following a raw vegan diet for four weeks [57].

In the OMN group, the highest mean values of LDL-C, non-HDL-C, and TC/HDL-C ratios compared to the other groups were observed. It should be pointed out that, according to the latest ESC/EAS guidelines [45], the non-HDL-C ratio is a vital risk factor [58,59,60].

Considering the lowest and highest mean values of the studied lipid profile parameters, the OMN group had the highest values of the parameters considered the main CV risk factors. On the other hand, in the VEGAN group with the lowest LDL-C and non-HDL-C levels, the lowest values of the CV risk parameters were observed. These observations are consistent with the results of other studies; see [48,51,56,61,62]. Another study on the Polish population during the COVID-19 pandemic, taking into account excessive body weight and the presence of abnormal lipid and carbohydrate metabolism values, showed a higher risk in the group of omnivores compared to the group of vegetarians [63].

#### 4.1.1. Apolipoproteins

Our study showed no statistically significant differences in apoB concentration between the analyzed groups. However, on average, the VEGAN group showed the highest concentration, and the lowest was observed in the PESCA group. It should be emphasized here that the ApoB measurement allows the estimation of atherogenic particles in plasma since this protein is a component of the LDL, VLDL, and TG-rich remnant particles [45]. According to the 2019 ESC/EAS guidelines, LDL-C, non-HDL-C, and ApoB levels are strongly associated. These parameters provide similar information on the risk of ASCVD in most cases [45,64,65,66].

In turn, the highest average values of ApoA were shown in the OMN group, then the VEGAN, and the lowest in the VEGE group. ApoA is the primary apolipoprotein of HDL, with ascribed anti-atherogenic potential; hence the higher the value of ApoA, the greater the protective effect against CV risk. It has to be mentioned that ApoA is also the protein part of other lipoproteins (VLDL, chylomicrons) [45], which may impact the results.

In the study by Dawczynski et al. [55], among the studied groups (omnivores, flexitarians, vegetarians, and vegans), the vegan group showed the lowest ApoA1 values (the flexitarian group showed the highest values). In the case of ApoB, the omnivores group showed the highest values. A statistically significant difference was established in the study by Bradbury et al. [61]; the ApoB values differed significantly between the groups among which the meat-eaters demonstrated the highest concentration, and the lowest concentration was demonstrated by vegans. Navarro et al. [62] showed a considerably higher ApoB in omnivores compared to vegetarians. In a study by Kuchta et al. [51], the omnivores and vegans’ groups significantly differed in the concentration of ApoB (0.69 vs. 0.54 g/L), which was higher in the omnivores group. At the same time, there were no significant differences between the ApoA concentration; the compared values were at the same level, 1.6 g/L.

The studies’ results most often revealed lower ApoA1 and ApoB values among vegans or vegetarians. Our results align with the lowest ApoA concentration in the VEGE group. However, the VEGAN group showed the second-highest average result for this parameter. On the other hand, concerning the concentration of ApoB, the results in the cited studies differ from ours. The lack of significant differences in the described results of the study population can be seen because they were mainly healthy and young people.

Taking into account the ApoB/ApoA1 ratio (known as the apo-ratio), indicating the balance between atherogenic and anti-atherogenic particles—the higher the score, the greater the CV risk [67]—the highest average values have been found in the VEGE group, then OMN (despite the average highest ApoA1 content) and VEGAN, and the lowest in the PESCA (with the lowest average ApoA1 value).

The highest score in the VEGE group is most likely due to their having the lowest average ApoA1 value for the general population and separately for men and women compared to the other groups. In the Adventist Health Study-2 [54], vegans and pesco-vegetarians had lower ApoB/ApoA ratio values than the non-vegetarian group, which is consistent with the results obtained in our study. On the other hand, the lacto-ovo-vegetarians got the lowest result of the described indicator, which differs from the results of our research. Our study showed another consistency with Adventist Health Study-2 [54] regarding the highest values in the non-vegetarian group of blood pressure, LDL-C, and non-HDL-C. Furthermore, in Dawczynski et.al. [55], omnivores showed the highest ApoB/ApoA1 score, significantly different from vegetarians and vegans (the lowest average value). In this study, the VEGAN group also showed a lower value than the OMN group, but these differences were not statistically significant.

#### 4.1.2. Lipoprotein (a)

The lipoprotein (a) concentration, described as a genetically determined biomarker of CV risk, in the studied groups was in line with the norm. Still, the OMN and VEGE groups showed the highest, on average, similar results, while the lowest were observed in the PESCA group (there were only trends, no abnormalities). In a study by Najjar et al. [57], a significant decrease in the concentration of lipoprotein (a) was observed after applying a vegan diet; it was the first study assessing the impact of the diet on this parameter. The VEGAN group showed a slightly lower concentration than the OMN group but higher than the PESCA group. More extensive studies based on a larger population would be indicated about the potential influence of diet on this parameter.

#### 4.1.3. Blood Pressure

According to the latest 2021 guidelines [1], it has been shown that raised BP is a significant cause of both ASCVD and non-atherosclerotic CVD. Our study demonstrated the highest values in the OMN group and the lowest in the PESCA. In studies comparing different types of vegetarian diets, the vegan group usually had the lowest blood pressure [54,68,69]; in our research, the VEGAN group ranked second (DBP) or third (SBP) in terms of the lowest values in comparison to other groups. There are also data from a meta-analysis [70,71] showing the therapeutic effect of vegan diets on blood pressure reduction.

#### 4.1.4. HCY

Increased concentration of HCY was considered a CV biomarker [72,73,74]. It should be noted that vitamin B12 deficiency can elevate HCY levels; hence, vegetarians, because of a possible B12 deficiency, more often than others, might have increased HCY [74]. The research results [74,75,76,77,78,79,80] showed higher concentrations of HCY in these groups, which is consistent with the results of our study. Still, it is necessary to emphasize that the population of this study showed only correct results with no statistical differences, which may indicate proper vitamin B12 supplementation among studied vegetarians and vegans.

### 4.2. Inflammatory Markers: hsCRP, IL-6, TNF-alpha

Recent studies have proposed that plant-based diets modulate the inflammatory markers’ profiles by attenuating them [81,82,83]. There is a need for further studies evaluating the link between vegetarian and vegan diets and biomarkers of inflammation, which will provide evidence on inflammation and its mechanisms, possibly related to plant-based diets, affecting the risk of numerous lifestyle diseases.

A meta-analysis by Menzel et al. [81] from 2020 has focused on the associations between vegan and vegetarian diets and inflammatory biomarkers compared to omnivores. A total of 21 cross-sectional studies were analyzed; among the involved parameters, there were hsCRP, IL-6, and TNF-alpha. The analyses revealed significant associations between CRP levels (including hsCRP) and vegan or vegetarian diet, with no substantial impact on other parameters.

#### 4.2.1. hsCRP

Only three studies [35,84,85] (data from [81]) have compared vegans and omnivores; the comparisons concerned CRP, including hsCRP in healthy participants. They have revealed significantly lower CRP levels (including hsCRP) in vegans (*p* < 0.0001) compared to omnivores, and this association was less pronounced in vegetarians (*p* = 0.05)). In a study conducted among vegans and omnivores, see [35], almost no significant differences were found if inflammatory biomarkers were taken into account. Only the omnivores group had significantly higher hsCRP levels (0.94  mg/l (95%-CI 0.65))−1.28)) compared to vegans (0.60 mg/L (95%-CI 0.36–0.87)). Regarding studies conducted among vegetarians, 14 studies among healthy people were included, involving 8 [86,87,88,89,90,91,92,93] that compare hsCRP levels and reveal lower CRP levels in vegetarians (*p* = 0.05). The results of another systematic review and meta-analysis [94], considering 30 studies, confirmed the CRP, including hsCRP, had significant differences between the groups. Another study also showed the possible impact of a vegan diet on a significant reduction in hsCRP levels [95]. These results are consistent with ours, where the VEGE and VEGAN groups showed slightly lower mean hsCRP values than the OMN and PESCA groups. However, these results were not statistically significant.

#### 4.2.2. TNF-Alpha

The meta-analysis [81], when focusing on TNF-alpha among healthy individuals who are either vegetarians or omnivores, disclosed three studies [85,93,96]: in two of them [85,93], vegetarians showed higher average TNF-alpha concentrations than omnivores; in the remaining one [96] the average result differed by only 0.01 pg/mL. All studies did not show significant statistical differences, which is consistent with our findings. The second-cited systematic review and meta-analysis [94] also showed no significant differences between the vegetarian-based and non-vegetarian groups. On the other hand, in another study [85], the CRP in vegetarians was higher than that of omnivores; this study also included a vegan group that showed the lowest mean CRP concentration, which is different if compared to the results of our research.

#### 4.2.3. IL-6

The meta-analysis [81] included the results of two studies [93,96] evaluating the concentration of IL-6 in healthy participants; in both cases, the concentration of IL-6 was higher in the vegetarian group, and in one of the studies [93], this difference was statistically significant.

These results differed from our study because the PESCA and OMN groups showed a higher IL-6 mean concentration than VEGE and VEGAN.

The correlations between the groups (Table 2) indicate similar results in the OMN and PESCA groups and the VEGE and VEGAN groups. An interesting repeatable result was a direct relation (positive correlation) between TNF-alpha and HCY concentration, which was observed in all study groups. It confirmed the results of another study’s observation among hypertensive patients [97]. In turn, in the study by Anghel et al. [98], the possibilities of the therapeutic use of anti-TNF-alpha and its impact on HCT levels in patients with rheumatoid arthritis (RA) were disclosed, and the need for broader research was emphasized.

### 4.3. Assessment of Possible Cardiovascular Risk by Creating Research Metrics

In this study, we have attempted to assess CV risk using various methods, including creating metrics considering multiple parameters.

The results showed no significant differences, only possible trends, by considering the average values, where the OMN group (along with PESCA in the metric result) showed the highest risk. In contrast, the VEGAN group had the lowest. Results of the 2021 Systematic Review of the Association Between Vegan Diets and Risk of Cardiovascular Disease [99] included over 73,000 participants, of whom at least 7661 were vegan; no study reported a significantly increased or decreased risk of any CV outcome. These results are consistent with the observations obtained in our study.

The differences between parameters in individual metrics (Table 4) are consistent with the observations in [81], such as increased risk with higher values of IL-6, HCY, or hsCRP.

The creation of four metrics taking into account different values of basic parameters (such as those included in risk cards) was aimed at revealing potential differences between healthy people aged below 40, using various diets. Furthermore, we checked whether new biomarkers could be helpful here. The obtained results were significant for ApoA1, IL-6, HCY, and hsCRP concentrations, which are related to CV risk estimates (based on the constructed metrics). It is consistent with recent reports that these markers have a positive predictive value for CV risk [81,100,101]. Moreover, each study group showed statistically significant results for a different biomarker concentration related to created metrics. Hence, it seems reasonable to recommend using more than one biomarker because there was no single biomarker result that would fit all participants. Our results indicated the need for further advanced research in this area.

The obtained results comply with global trends indicating the need to reduce the consumption of meat products in favor of increasing plant-based products and, above all, improving the quality of nutrition worldwide. It has been documented that people who limit or eliminate animal products suffer from CVD to a lesser extent [28,61,70,102,103,104]. In addition, numerous studies have confirmed the impact of plant-based diets on health [28,105,106,107,108], showing a link between this diet and a lower risk of cancer, obesity, diabetes, and hypertension.

By definition, giving up meat consumption results in a lower supply of saturated fatty acids, but nowadays, where access to ready-made, processed vegetarian products is broad, these diets may also be characterized by the share of foods rich in saturated fatty acids, trans fatty acids, sugar, and salt. The positive impact of a vegetarian diet accompanied by highly processed products will be leveled, as shown by the Nurses’ Health Study [109], which considers the effects on CV risk when using healthful or unhealthful plant-based diets as the quality of the diet was checked. Other research from the Nurses’ Health Study also shows that adherence to a healthy plant-based diet may reduce the risk of breast cancer [110], developing type 2 diabetes [111], or stroke [112].

Some parameters could be prognostic, allowing estimating the 10–20-year risk of CV diseases already at a younger age, or it may show the need for intervention in one of the examined nutritional groups or indicate the type of diet with the most significant advantages. Our study attempted such a search.

## 5. Strengths and Limitations of the Study

### 5.1. Strengths of the Study

- To the best of our knowledge, this is the first study comparing lipids, lipoproteins, and other CV parameters and numerous inflammatory markers among healthy people <40 being on different diets.
- The study was conducted during the COVID-19 pandemic. However, compared to other studies, the number of respondents is relatively high, considering how unique groups were recruited.
- The results of this study confirm the possibly beneficial consequences on cardiovascular risk of using a well-balanced vegan diet and maybe another argument “in favor” of recommending this diet. Specialists in various fields of medicine may consider recommending their use in some instances.
- The study participants did not show differences in average age, which proves the samples’ reliability. It was initially planned to divide the study participants into age categories, but due to the above, such division was abandoned.

### 5.2. Limitations of the Study

- The number of women and men in the study groups is random; the respondents are mainly women.
- The narrow age group does not provide a complete cross-section of possible outcomes.
- Due to the subjects’ young age and health status, comparisons were not made concerning CV risk using the standard recommended CV risk factors tools, which mainly consider age 40 and above. The lack of direct differences concerning diagnostic indications may result from a young age and targeted selection of healthy people.
- The blood sampling assessment as a diagnostic part of the study was performed once. This is a single sample study due to the timing of the pandemic.
- Possible mistakes may have resulted from the incorrect preparation of the participants, or other errors from the pre-analytical stage, as well as during the analyses performed, and these may have led to unintentional biases.
- The criterion of vaccination against SARS-CoV-2 (severe acute respiratory syndrome coronavirus-2) was not a necessary condition for entering the study. The study was conducted during the COVID-19 pandemic; there is a risk that some of the subjects may have been infected (without symptoms), which could have affected the obtained results; however, the obtained results (within the normal range) exclude the influence of the disease.

## 6. Conclusions

The results of this study indicate that the prediction of CV risk among healthy people under 40 years of age with various dietary patterns, using selected biochemical parameters and created indicators, is difficult but feasible. The metrics creation significantly contributed to obtaining a predictive outcome for the studied groups, demonstrating repetitive patterns. In addition, the results confirmed the feasibility of using new indicators such as ApoA1, IL-6, HCY, and hsCRP to measure CV risk.

Moreover, the obtained results allow for extending the current knowledge on the possible impact of vegetarian diets, including vegan ones, on health.

The conducted study can be an indication for further, broader research on the search for CV risk biomarkers in healthy people under 40, still showing only regularities in the basic assessments. This group is extremely important because, here, most of the habits are formed and impact future health.

## Figures and Tables

**Table 1:** Baseline laboratory characteristics.

**Variables**	**Sex**	**Studied Groups n = 155**				*p* *
		**OMN** *n* = 35 F/M: 21/14 %F 60.0	**PESCA** *n* = 23 F/M: 20/3 %F 86.9	**VEGE** *n* = 49 F/M: 45/4 %F 91.8	**VEGAN** *n* = 48 F/M: 32/16 %F 66.7	
**Blood lipids and lipoproteins** mean ± SD (min–max)						
**ApoA1** **[mg/mL]**	All	2.14 ± 1.77 (0.458–6.11)	1.56 ± 1.09 (0.459–4.26)	1.30 ± 0.948 (0.248–5.69)	1.80 ± 1.87 (0.269–7.26)	0.380
	F	2.17 ± 1.78 (0.548–6.11)	1.64 ± 1.15 (0.459–4.26)	1.33 ± 0.980 (0.248–5.69)	1.92 ± 2.01 (0.459–7.26)	0.561
	M	2.09 ± 1.83 (0.458–6.11)	1.05 ± 0.151 (0.875–1.15)	0.946 ± 0.317 (0.615–1.35)	1.55 ± 1.59 (0.269–6.11)	0.608
**ApoB** **[mg/mL]**	All	1.05 ± 0.744 (0.252–3.36)	0.957 ± 0.495 (0.381–2.04)	1.04 ± 0.700 (0.252–3.96)	1.15 ± 0.983 (0.252–3.96)	0.983
**ApoB/ApoA1**	F	0.914 ± 1.28 (0.0656–7.34)	0.871 ± 0.811 (0.115– 4.05)	0.944 ± 0.684 (0.186–4.01)	0.900 ± 1.06 (0.0730–6.85)	0.107
	M	0.763 ± 0.680 (0.0656–2.82)	0.873 ± 0.859 (0.115–4.05)	0.975 ± 0.702 (0.207–4.01)	0.805 ± 0.669 (0.0730–4.07)	0.172
	All	1.14 ± 1.87 (0.0667–7.34)	0.852 ± 0.461 (0.545–1.38)	0.600 ± 0.290 (0.186–0.862)	1.09 ± 1.60 (0.337–6.85)	0.820
**Lp(a)** **[mg/dL]**	All	23.8 ± 18.0 (5.80–74.1)	19.1 ± 16.1 (6.62–79.2)	23.6 ± 17.4 (2.70–81.1)	22.5 ± 17.6 (9.30–71.4)	0.404
**TC** **[mg/dL]**	All	158 ± 30.3 (86–207)	160 ± 34.7 (93–218)	157 ± 22.8 (113–220)	142 ± 29.2 (71–206)	0.032
**HDL–C** **[mg/dL]**	All	55.8 ± 14.3 (36–92)	60.6 ± 13.0 (41–94)	59.3 ± 13.4 (26–94)	51.7 ± 10.1 (31–72)	0.006
	F	60.8 ± 15.6 (36–92)	61.9 ± 13.0 (44–94)	60.7 ± 12.9 (26–94)	53.4 ± 10.6 (31–72)	0.055
	M	48.4 ± 8.04 (37–62)	52.0 ± 11.0 (41–63)	43.8 ± 6.99 (37–52)	48.3 ± 8.43 (35–70)	0.607
**LDL–C** **[mg/dL]**	All	80.1 ± 20.9 (30–113)	75.1 ± 24.8 (39.6–136)	74.1 ± 18.2 (32–139)	70.0 ± 21.0 (21–111)	0.133
**TG** **[mg/dL]**	All	77.9 ± 34.7 (33–202)	92.4 ± 45.9 (44–217)	94.0 ± 27.2 (49–153)	95.1 ± 26.4 (41–165)	0.005
**Non–HDL–C [mg/dL]**	All	102 ± 27.9 (47–153)	99.6 ± 35.0 (39–155)	98.1 ± 19.9 (64–158)	90.1 ± 25.9 (28–149)	0.297
**TC/HDL–C**	All	2.94 ± 0.778 (1.74–5.03)	2.73 ± 0.698 (1.54–3.78)	2.76 ± 0.613 (1.83–4.96)	2.79 ± 0.596 (1.65–4.37)	0.746
**Other blood parameters** mean ± SD (min–max)						
**HCY** **[nmol/mL]**	All	10.0 ± 5.53 (4.10–33.7)	15.3 ± 8.78 (5.87–33.7)	12.8 ± 8.17 (5.07–37.9)	14.3 ± 10.9 (4.06–37.9)	0.096
**TNF–alpha [ng/L]**	All	89.1 ± 39.2 (52.8–193	111 ± 49.1 (54.1–203)	121 ± 83.8 (51.5–368)	128 ± 90.6 (52.8–368)	0.149
**hsCRP** **[mg/L]**	All	5.19 ± 3.01 (0.564–11.2)	5.47 ± 3.45 (1.12–15.7)	3.83 ± 2.50 (0.675–9.65)	4.76 ± 3.07 (0.962–13.7)	0.100
**GLU** **[mg/dL]**	All	88.1 ± 10.3 (60.1–107)	85.4 ± 9.49 (65.8–98)	88.4 ± 6.04 (75.2–101)	87.7 ± 8.19 (59.3–105)	0.658
**UA** **[mg/dL]**	All	4.41 ± 1.40 (1.46–6.77)	4.21 ± 1.52 (1.29–8.64)	4.54 ± 0.877 (2.76–7.22)	4.85 ± 0.986 (2.57–7.28)	0.057
**IL–6** **[pg/mL]**	All	3.05 ± 1.87 (0.367–6.91)	3.16 ± 2.40 (0.131–7.09)	2.87 ± 2.31 (0.341–8.54)	2.79 ± 2.27 (0.341–8.12)	0.833
**Blood pressure and pulse** mean ± SD (min–max)						
**SBP** **[mm/Hg]**	All	122 ± 14.5 (97–153)	116 ± 12.2 (95–143)	118 ± 9.68 (99–140)	119 ± 11.1 (96–153)	0.439
	F	117 ± 12.3 (97–151)	115 ± 12.6 (95–143)	118 ± 9.47 (99–140)	116 ± 9.99 (96–136)	0.490
	M	129 ± 14.9 (107–153)	122 ± 6.03 (116–128)	121 ± 13.2 (104–136)	125 ± 11.6 (104–153)	0.788
**DBP** **[mm/Hg]**	All	76 ± 8.27 (60–96)	71.9 ± 7.83 (57–82)	75.4 ± 6.62 (60–92)	74.5 ± 8.51 (52–89)	0.376
	F	75.4 ± 8.81 (60–96)	71.0 ± 7.89 (57–82)	75.3 ± 6.73 (60–92)	74.5 ± 8.45 (52–86)	0.303
	M	76.9 ± 7.63 (66–89)	77.7 ± 5.13 (72–82)	75.8 ± 6.13 (70–82)	74.4 ± 8.90 (62–89)	0.727
**Pulse** **[bpm]**	All	78.0 ± 15.7 (46–121)	74.6 ± 14.5 (56–104)	82.5 ± 13.5 (52–117)	77.0 ± 14.0 (40–114)	0.053
	F	80.8 ± 13.7 (51–112)	75.0 ± 14.8 (56–104)	82.6 ± 13.6 (52–117)	80.3 ± 15.1 (40–114)	0.229
	M	73.8 ± 17.9 (46–121)	71.3 ± 15.5 (56–87)	81.5 ± 13.7 (61–89)	70.7 ± 9.00 (53–83)	0.359

* *p* was calculated to assess differences between groups concerning selected variables, including separately for gender; *p* < 0.05 considered statistically significant. Abbreviations: F—Female, M—Male, ApoA1—Apolipoprotein A1, ApoB—Apolipoprotein B, ApoB/ApoA1—Apolipoprotein B/apolipoprotein A1 ratio, Lp(a)—Lipoprotein(a), TC—Total cholesterol, HDL-C—High-density lipoprotein cholesterol, LDL-C—Low-density lipoprotein cholesterol, TG—Triglycerides, Non-HDL-C—Non-high-density lipoprotein cholesterol, TC/HDL-C—Total cholesterol to high-density lipoprotein cholesterol ratio, HCY—Homocysteine, TNF-alpha—Tumor necrosis factor alpha, hsCRP—High sensitivity C-reactive protein, GLU—Glucose, UA—Uric acid, IL-6—Interleukin 6, SBP—Systolic blood pressure, DBP—Diastolic blood pressure, bpm—beats per minute.

**Table 2:** The strongest correlations between the parameters in the study groups.

Group	The Strongest Correlations between the Studied Parameters *p* < 0.001
OMN (for detailed data, see Table S2)	TG and GLU (positive correlation) TNF-alpha with HCY (positive correlation); TNF-alpha with sleep duration (negative correlation) VAT and BFM, Body mass, BMI, PBF, WHR, waist and hips circuit (positive correlations) *
PESCA (for detailed data, see Table S3)	TG and GLU (positive correlation) TNF-alpha and HCY (positive correlation) VAT and BFM, body mass, BMI, PBF, WHR, waist and hips circuit (positive correlations) *
VEGE (for detailed data, see Table S4)	ApoA1 and TNF-alpha, HCY (positive correlations) ApoB and TNF-alpha, HCY, Lp(a) (positive correlations) TNF-alpha and HCY, Lp(a) (positive correlations) HCY and Lp(a) (positive correlation) VAT and BFM, body mass, BMI, PBF, WHR, waist and hips circuit (positive correlation) *
VEGAN (for detailed data, see Table S5)	ApoA1 and TNF-alpha, HCY, Lp(a) (positive correlations) ApoB and TNF-alpha, HCY, Lp(a) (positive correlations) HCY and Lp(a) (positive correlation) VAT and BFM, BMI, PBF, WHR, waist and hips circuit, physical activity, DBP (positive correlations) *

* The body composition results (BMI, VAT, BFM, PBF, WHR) of all studied groups were within the normal ranges and showed no significant differences between the studied groups; the SMM analysis showed significant differences (the highest mean value was observed in OMN group, and the lowest in VEGE group; the VEGAN group showed one of the highest values (next to the OMN group). These parameters were measured using the BIA method [38]. Abbreviations: BFM—Body Fat Mass; BMI—Body Mass Index; PBF—Percentage Body Fat; SMM—Skeletal Muscle Mass; WHR—Waist-Hip Ratio; VAT—Visceral Adipose Tissue; Sleep duration (h), and Physical activity (low = 1, medium = 2, high = 3) were calculated at the first stage of the study [38]; ApoA1—Apolipoprotein A1, ApoB—Apolipoprotein B, Lp(a)—Lipoprotein(a), TG—triacylglycerols, HCY—Homocysteine, TNF-alpha—Tumor necrosis factor alpha, hsCRP—High sensitivity C-reactive protein, GLU—glucose, DBP—Diastolic blood pressure.

**Table 3:** Trial for estimating CV by use of metrics.

	METRIC 1	METRIC 2	METRIC 3	METRIC 4
Parameters included in metrics	SBP > 140 mm/Hg low physical activity TC > 190 mg/dL smoking * family burden of CVD * family burden of lipid metabolism abnormalities * family burden of carbohydrate abnormalities *	SBP > 140 mm/Hg TC > 190 mg/dL HDL-C < 50 mg/dL in female, and < 40 mg/dL in male smoking *	SBP > 140 mm/Hg TC > 190 mg/dL HDL-C < 50 mg/dL in female, and < 40 mg/dL in male LDL-C > 115 mg/dL TG > 150 mg/dL GLU > 99 mg/dL UA > 5.7 mg/dL in female, and > 7.0 mg/dL in male smoking * family burden of CVD * family burden of lipid metabolism abnormalities * family burden of carbohydrate abnormalities *	TC > 190 mg/dL HDL-C < 50 mg/dL in female, and < 40 mg/dL in male LDL-C > 115 mg/dL TG > 150 mg/dL smoking * family burden of CVD * family burden of lipid metabolism abnormalities * family burden of carbohydrate abnormalities *
**GROUP**	**Mean ± SD**			
	*p-*value			
OMN	1.71 ± 1.23	0.571 ± 0.698	1.97 ± 1.40	1.86 ± 1.29
PESCA	1.57 ± 1.24	0.435 ± 0.590	1.91 ± 1.62	1.87 ± 1.52
VEGE	1.45 ± 1.17	0.327 ± 0.474	1.69 ± 1.19	1.61 ± 1.20
VEGAN	1.42 ± 1.16	0.354 ±0.483	1.69 ± 1.24	1.54 ± 1.05
*p* *^1^	0.723	0.388	0.757	0.619
OMN + PESCA	1.66 ± 1.22	0.517 ± 0.655	1.95 ± 1.48	1.86 ± 1.37
VEGE + VEGAN	1.43 ± 1.16	0.340 ± 0.476	1.69 ± 1.21	1.58 ± 1.13
*p* *^2^	0.155	0.061	0.170	0.103

* Information regarding smoking, the family burden of CVD, and lipid metabolism or carbohydrate abnormalities come from the questionnaire (see Supplementary Materials Questionnaire S1). The results regarding smoking frequency did not show statistical significance between the study groups [38]. *^1^ *p*—calculated to assess whether there were differences between all the studied groups using Kruskal–Wallis (One-Way ANOVA) Test; *^2^ *p*—calculated to assess whether there were differences between VEGE + VEGAN and OMN + PESCA using Mann–Whitney U Test with hypothesis μ_VEGE + VEGAN_ < μ_OMN + PESCA_. *p* < 0.05 considered statistically significant. Abbreviations: SBP—Systolic blood pressure, TC—Total cholesterol, HDL-C—High-density lipoprotein cholesterol, LDL-C—low-density lipoprotein cholesterol, TG—Triacylglycerols, GLU—Glucose, UA—Uric acid, CVD—Cardiovascular disease.

**Table 4:** Relationships between the metric estimate of cardiovascular risk and the biochemical parameters in the studied groups. The data included only statistically significant results.

Group		
Metric	Parameter	p **
**OMN**		
METRIC 1 (group L = 0–1 group H > 1) *	**ApoA1 [mg/mL]** H 1.809 vs. L 2.632 median: H 0.945 vs. L 1.797	0.028
**PESCA**		
METRIC 1 (group L = 0–1 group H > 1) *	**IL-6 [pg/mL]** H 3.988 vs. L 2.401 median: H 4.098 vs. L 1.888	0.046
**VEGAN**		
METRIC 3 (group L = 0–1 group H > 1) *	**HCY [nmol/mL]** H 16.444 vs. L 12.009 median: H 10.624 vs. L 8.611	0.050
**OMN + PESCA**		
METRIC 2 (group L = 0 group H > 0) *	**hsCRP [mg/L]** H 6.269 H vs. L 4.509 median: H 5.942 vs. L 4.216	0.025

* according to results from Table 3. ** Mann–Whitney U Test with the one-tailed hypothesis. Abbreviations: ApoA1—Apolipoprotein A1, IL-6—Interleukin 6, HCY—Homocysteine, hsCRP—High sensitivity C-reactive protein

## Data Availability

All necessary data are included in the paper.

## Supplementary Materials

The following supporting information can be downloaded at https://www.mdpi.com/article/10.3390/ijerph20032237/s1, Questionnaire S1: The cardiovascular risk burden questionnaire; Table S1: Biochemical methods and reference ranges; Tables of Correlations (including Tables S2–S5).
