# Supplementary material for: Is It Feasible to Predict Cardiovascular Risk among Healthy Vegans, Lacto-/Ovo-Vegetarians, Pescatarians, and Omnivores under Forty?
Source: Int J Environ Res Public Health. 2023 Jan 27;20(3):2237. doi: 10.3390/ijerph20032237 (PMC9915557; doi:10.3390/ijerph20032237)
Supplement: Supplementary file 1 [file ijerph-20-02237-s001.zip › Questionnaire S1.pdf]

Supplementary Materials:

Questionnaire S1: The cardiovascular risk burden questionnaire.

1) Do you smoke cigarettes?
   a) yes, occasionally (about 1-3 cigarettes per week)
   b) yes, regularly small amounts (less than a pack a day)
   c) yes, regularly large amounts (more than a pack a day)
   d) do not smoke

2) Among first-degree relatives (parents, siblings), have there been/are there any cardiovascular diseases, i.e. heart disease, heart attack, stroke, thrombosis (in men before the age of 55, in women before the age of 60) ?
 a) yes
 b) no

3) Did/does your first-degree relatives (parents, siblings, children) have abnormal lipid profile values (total cholesterol, triglycerides, HDL,LDL fraction cholesterol) ?
   a) yes
   b) no

4) Among first-degree relatives (parents, siblings, children) were/are there abnormal fasting glucose values, or relatives have diabetes/insulin resistance ?
   a) yes
   b) no
