# Supplementary material for: Is It Feasible to Predict Cardiovascular Risk among Healthy Vegans, Lacto-/Ovo-Vegetarians, Pescatarians, and Omnivores under Forty?
Source: Int J Environ Res Public Health. 2023 Jan 27;20(3):2237. doi: 10.3390/ijerph20032237 (PMC9915557; doi:10.3390/ijerph20032237)
Supplement: Supplementary file 1 [file ijerph-20-02237-s001.zip › Tables of Correlations(Tables S2-S4).pdf]

Supplementary Materials: Tables S2 – S5

Table S2. Correlations between biochemical parameters, body composition parameters and behavioral factors in OMN group.

Groups	Parameter 1	Parameter 2	Spearman's ρ	*p*
OMN	ApoA1	LDL-C	-0.356	0.036
	TC	HCY	-0.460	0.005
		GLU	0.496	0.003
		Pulse	0.473	0.004
	HDL-C	TG	0.510	0.002
		UA	-0.439	0.008
		TBW	-0.414	0.013
		Minerals	-0.364	0.031
		Body mass	-0.347	0.041
		Muscle mass	-0.424	0.011
		Sleep duration	0.339	0.046
		Waist circuit	-0.346	0.041
	LDL-C	TNF-alpha	-0.337	0.048
		HCY	-0.426	0.011
		Pulse	0.485	0.003
		TG	0.430	0.010
	TG	UA	0.468	0.005
		GLU	0.692	< 0.001
		TBW	0.349	0.040
		BMI	0.345	0.042
		Muscle mass	0.358	0.034
		Waist circuit	0.446	0.007
	TNF-alpha	HCY	0.797	< 0.001
		Sleep duration	-0.621	< 0.001
	HCY	Sleep duration	-0.454	0.006
	UA	GLU	0.473	0.005
		TBW	0.369	0.029
		Muscle mass	0.375	0.027
	IL-6	BFM	0.368	0.030
		Sleep duration	0.336	0.048
		Hips circuit	0.383	0.023
		VAT	0.419	0.012
	VAT	Minerals	0.336	0.048
		BFM	0.972	< 0.001
		Body mass	0.542	< 0.001
		BMI	0.727	< 0.001
		PBF	0.775	< 0.001
		WHR	0.623	< 0.001
		Waist circuit	0.554	< 0.001
		Hips circuit	0.542	< 0.001
		DBP	0.393	0.019

Table S3. Correlations between biochemical parameters, body composition parameters and behavioral factors in in PESCA group.

Groups	Parameter 1	Parameter 2	Spearman's ρ	*p*
PESCA	ApoB	TNF-alpha	0.420	0.046
		HCY	0.538	0.008
	TC	UA	0.514	0.012
		GLU	0.463	0.026
		TG	0.587	0.003
	HDL-C	Physical activity	0.432	0.039
		IL-6	-0.437	0.037
		Lp(a)	0.507	0.014
	TG	UA	0.550	0.007
		GLU	0.421	0.045
		Pulse	0.415	0.049
		TBW	0.580	0.004
		Minerals	0.480	0.020
		Body mass	0.481	0.020
		Muscle mass	0.572	0.004
		WHR	0.417	0.048
	TNF-alpha	HCY	0.761	< 0.001
	hsCRP	BMI	0.418	0.047
	UA	LDL-C	0.509	0.013
		TBW	0.437	0.038
		Muscle mass	0.435	0.038
	VAT	TBW	0.447	0.032
		Minerals	0.555	0.006
		BFM	0.978	< 0.001
		Body mass	0.764	< 0.001
		BMI	0.896	< 0.001
		PBF	0.872	< 0.001
		Muscle mass	0.435	0.038
		WHR	0.768	< 0.001
		Waist circuit	0.764	< 0.001
		Hips circuit	0.818	< 0.001

Table S4. Correlations between biochemical parameters, body composition parameters and behavioral factors in in VEGE group.

Groups	Parameter 1	Parameter 2	Spearman's ρ	*p*
VEGE	ApoA1	TNF-alpha	0.646	< 0.001
		HCY	0.653	< 0.001
		Lp(a)	0.431	0.002
		HDL-C	0.283	0.049
		BFM	0.375	0.008
		BMI	0.348	0.014
		WHR	0.408	0.004
		Waist circuit	0.380	0.007
		Hips circuit	0.333	0.019
		VAT	0.301	0.036
	ApoB	TNF-alpha	0.552	< 0.001
		HCY	0.560	< 0.001
		Lp(a)	0.794	< 0.001
	TC	HCY	0.352	0.013
		Sleep duration	0.296	0.039
		Waist circuit	0.288	0.045
	HDL-C	Phisical activity	0.382	0.007
	LDL-C	HCY	0.307	0.032
		Sleep duration	0.303	0.034
	TG	Waist circuit	0.391	0.006
		DBP	0.320	0.025
	TNF-alpha	HCY	0.786	< 0.001
		Lp(a)	0.475	< 0.001
		Waist circuit	0.301	0.036
	HCY	Lp(a)	0.507	< 0.001
		PBF	0.288	0.045
	Lp(a)	BFM	0.387	0.006
		BMI	0.293	0.041
		PBF	0.399	0.005
	UA	GLU	0.293	0.041
	IL-6	Body mass	0.284	0.048
		WHR	0.293	0.041
		SBP	0.300	0.036
		VAT	0.304	0.034
	VAT	Minerals	0.287	0.046
		BFM	0.924	< 0.001
		Body mass	0.626	< 0.001
		BMI	0.622	< 0.001
		PBF	0.692	< 0.001
		WHR	0.734	< 0.001
		Waist circuit	0.613	< 0.001
		Hips circuit	0.540	< 0.001

Table S5. Correlations between biochemical parameters, body composition parameters and behavioral factors in in VEGAN group.

Groups	Parameter 1	Parameter 2	Spearman's ρ	*p*
VEGAN	ApoA1	TNF-alpha	0.493	< 0.001
		HCY	0.619	< 0.001
		Lp(a)	0.463	< 0.001
	ApoB	TNF-alpha	0.605	< 0.001
		HCY	0.676	< 0.001
		Lp(a)	0.611	< 0.001
	LDL-C	TG	0.306	0.035
	TG	BFM	0.329	0.022
		DBP	0.314	0.030
		VAT	0.325	0.024
	TNF-alpha	HCY	0.779	< 0.001
		Lp(a)	0.395	0.005
	HCY	Lp(a)	0.540	< 0.001
	UA	TBW	0.302	0.037
		Muscle mass	0.304	0.036
	IL-6	BFM	0.328	0.023
		Physical activity	-0.451	0.001
		Waist circuit	0.327	0.023
		Hips circuit	0.337	0.019
		VAT	0.309	0.033
		Pulse	0.296	0.043
	VAT	BFM	0.976	< 0.001
		Body mass	0.326	0.024
		BMI	0.602	< 0.001
		PBF	0.884	< 0.001
		WHR	0.511	< 0.001
		Physical activity	-0.460	0.001
		Waist circuit	0.634	< 0.001
		Hips circuit	0.620	< 0.001
		DBP	0.508	< 0.001

Abbreviations: BFM—Body Fat Mass; BMI—Body Mass Index; PBF—Percentage Body Fat; SMM—Skeletal Muscle Mass; WHR—Waist-Hip Ratio; VAT—Visceral Adipose Tissue, Sleep duration (h) and Physical activity (low = 1, medium = 2, high = 3) was calculated at the first stage of the study, and ApoA1 – Apolipoprotein A1, ApoB – Apolipoprotein B, ApoB/ApoA1 – apolipoprotein B / apolipoprotein A1 ratio, Lp(a) – Lipoprotein(a), TC – Total cholesterol, HDL-C – High-density lipoprotein cholesterol, LDL-C – Low-density lipoprotein cholesterol, TG – Triacylglycerols, Non-HDL-C – Non-high-density lipoprotein cholesterol, TC/HDL-C – Total cholesterol to high-density lipoprotein cholesterol ratio, HCY – Homocysteine, TNF-alpha – Tumour necrosis factor alpha, hsCRP – High sensitivity C-reactive protein, GLU – Glucose, UA – Uric acid, IL-6 – Interleukin 6, SBP – Systolic blood pressure, DBP – Diastolic blood pressure.
